# Immobilization-Free Electrochemical Sensor Coupled with a Graphene-Oxide-Based Aptasensor for Glycated Albumin Detection

**DOI:** 10.3390/bios11030085

**Published:** 2021-03-17

**Authors:** Wassa Waiwinya, Thitirat Putnin, Dechnarong Pimalai, Wireeya Chawjiraphan, Nuankanya Sathirapongsasuti, Deanpen Japrung

**Affiliations:** 1 National Nanotechnology Center (NANOTEC), National Science and Technology Development Agency (NSTDA), Thailand Science Park, Pathumthani 12120, Thailand; wassa.waiwinya@gmail.com (W.W.); thitirat.put@ncr.nstda.or.th (T.P.); dechnarong.pim@nanotec.or.th (D.P.); wireeya.cha@nanotec.or.th (W.C.); 2 Section of Translational Medicine, Faculty of Medicine Ramathibodi Hospital, Mahidol University, Bangkok 10400, Thailand; nuankanya@gmail.com

**Keywords:** graphene oxide, glycated human serum albumin, immobilization-free electrochemical measurement, screen-printed carbon electrode, diabetes mellitus

## Abstract

An immobilization-free electrochemical sensor coupled with a graphene oxide (GO)-based aptasensor was developed for glycated human serum albumin (GHSA) detection. The concentration of GHSA was monitored by measuring the electrochemical response of free GO and aptamer-bound GO in the presence of glycated albumin; their currents served as the analytical signals. The electrochemical aptasensor exhibited good performance with a base-10 logarithmic scale. The calibration curve was achieved in the range of 0.01–50 µg/mL. The limit of detection (LOD) was 8.70 ng/mL. The developed method was considered a one-drop measurement process because a fabrication step and the probe-immobilization process were not required. This simple sensor offers a cost-effective, rapid, and sensitive detection method, and could be an alternative approach for determination of GHSA levels.

## 1. Introduction

Diabetes mellitus (DM) is a metabolic disease caused by the presence of high blood glucose levels or hyperglycemia resulting from impaired insulin secretion, defective insulin action, or both [1,2]. According to the World Health Organization (WHO), approximately 422 million people worldwide are diabetic, with a particularly high incidence in low- and middle-income countries; 1.6 million deaths are directly attributed to diabetes each year. The complications associated with diabetes, such as neuropathy, nephropathy, retinopathy, and cardiovascular disease, which arise in both type 1 and type 2 diabetes, are core factors of severe morbidity, mortality, and huge economic burdens [1,2,3,4,5,6,7,8]. Therefore, screening at an early stage is important for the management of patients with diabetes. Currently, the WHO recommends evaluation of blood glucose levels and glycated hemoglobin (HbA1c) as screening and diagnostic criteria for diabetes [9]. However, both biomarkers have limitations. For blood glucose, fasting for at least 8 h is required. Measurement of HbA1c levels is generally inaccurate in patients with erythropoiesis, genetic blood disorders (thalassemia, anemia, or abnormal hemoglobin), and chronic kidney disease (CKD) [10,11]. In recent studies, glycated human serum albumin (GHSA), which is a nonenzymatic glycation product of albumin, has been reported as a biomarker for the screening and monitoring of DM when rapid treatment responses are required because of the shorter lifespan of serum albumin (half-life of 17 days) [12,13]. GHSA has been proposed as a biomarker for glycemic control in patients with anemia, pregnancy, abnormal hemoglobin, and diabetes accompanied by CKD [8,14,15,16]. Considering the importance of GHSA levels in clinical samples, the development of accurate and efficient analytical approaches for GHSA measurement is required.

Various techniques, including chromatography, immunoassay, high-performance liquid chromatography, Raman spectroscopy, capillary electrophoresis, and fluorescence- and electrochemical-based aptasensors, have been developed to examine the presence of GHSA [15,16,17,18,19]. Among these techniques, electrochemical platforms have attracted much attention because of their high sensitivity, rapid evaluation protocols, and cost effectiveness. Recently, Bunyarataphan et al. reported the use of a superficial electrochemical aptasensor to detect GHSA in normal and DM serum samples. Although the developed platform is sensitive, the immobilization step is time consuming and requires an excess amount of probe [20].

In the past decade, interest has increased in exploring graphene and its derivatives, such as graphene oxide (GO), for modification of electrode surfaces to enhance the sensitivity of electrochemical related platforms, owing to the large surface area, electrical conductivity, and high electron transfer rate of these molecules, as well as their capacity to immobilize various molecules and facilitate chemical processing [21]. In particular, graphene is capable of adsorbing oligonucleotide by forming strong π-π interactions with the nitrogenous bases of the nucleotides. Hence, graphene is being increasingly applied as a transducing enhancement for biosensing, particularly in medical and biological applications [22,23]. The high sensitivity, low production cost, simplicity, and short analysis time of the sensor are the main requirements for sensor development.

In this study, we aimed to improve the performance of the electrochemical aptasensor, particularly in terms of sensitivity and simplicity. To this end, an electrochemical aptasensor using a GO-based approach was developed. The aptasensor solutions, which contained the GO-aptamer complex, protein target, and electrolyte, were drop-cast onto a screen-printed carbon electrode (SPCE), and the electrochemical signal was immediately measured. This strategy overcame the disadvantages of the immobilization step and the complexity of the fabrication process. This study illustrates the proof-of-concept of a single-drop measurement process for electrochemical sensors based on GO for GHSA detection, supporting the use of this platform as an alternative method for determining GHSA levels in clinical samples.

## 2. Materials and Methods

### 2.1. Chemicals and Materials

The modified G8-FAM (G8-fluorescein) and modified G8 aptamer-bound glycated albumin were chemically synthesized and purchased from Integrated DNA Technologies, Inc. (Iowa, USA). The nucleotide sequences are listed in Table 1. GHSA, human serum albumin (HSA), immunoglobulin G (IgG), hemoglobin (Hb), potassium chloride, ethanolamine, and potassium ferricyanide were purchased from Sigma-Aldrich Co. (Missouri, USA). The GHSA protein was purified from human serum using chilled ethanol, followed by chromatographic methods and in vitro glycation. Then, the protein purity was evaluated by sodium dodecyl sulfate-polyacrylamide gel electrophoresis (SDS-PAGE). Working solutions of ethanolamine were prepared in deionized (DI) water. The aptamer stock solutions (2.0 µM) were prepared with sterile deionized water and kept at −20 °C. SPCEs were purchased from Zensor (Taichung, Taiwan). All reagents were of analytical grade.

### 2.2. Preparation of Electrodes

SPCEs consisting of a 3 mm diameter carbon working electrode, counter, and reference electrodes were used as electrochemical transducers in conjunction with a specific cable connector. The electrode surface was treated with 20 µL of 0.05 M ethanolamine for 20 min [20] and then washed in phosphate-buffered saline (PBS; pH 7.4) for 30 s at room temperature with gentle stirring.

### 2.3. Preparation of the GO-Aptasensor Complex

GO was prepared using a modified Hummers’ method, as described in our previous study [19]. Then, GO powder was dissolved in sterile water to make a 5.0 mg/mL solution that was sonicated for at least 15 min to produce a homogeneous stock solution. The GO stock solution was dissolved in DI water with gentle mixing for at least 5 s to obtain a working concentration of 2 mg/mL. To prepare the GO-aptasensor complex solution, 5 µL of 2.0 mg/mL GO was mixed with 5 µL of 2 µM modified G8 aptamer and incubated at room temperature in the dark. After 5 min of incubation, the GO-aptasensor complex solution was used for GHSA detection in both electrochemical and fluorescence measurements. The structural morphologies of the free GO and the electrode in the presence and absence of GO were also studied using scanning electron microscopy (SEM) and transmission electron microscopy, as described in the Supplementary Materials (Figures S1 and S2).

### 2.4. Electrochemical Measurement

**Figure 1:**
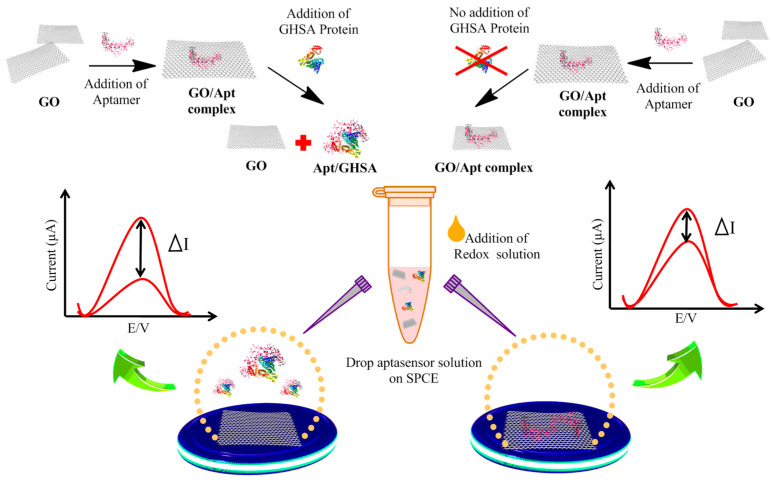
Schematic illustration of the immobilization-free electrochemical sensor coupled with the graphene oxide (GO)-based aptasensor for glycated human serum albumin (GHSA) detection. In the presence of GHSA, the aptamer detaches from the GO surface and specifically binds to GHSA, leading to adsorption of free GO on the electrode surface. In the absence of GHSA, the aptamer is still attached on the GO surface, leading to adsorption of the GO-aptamer complex on the electrode surface. After square wave voltammetry (SWV) determination, the current change (ΔI) is calculated and plotted against the GHSA concentration.

The electrochemical characterization and determination of GHSA using the GO-aptasensor complex solution were carried out on an Autolab PGSTAT302N potentiostat/galvanostat (Eco Chemie, Utrecht, The Netherlands) and controlled with Autolab NOVA software version 1.10. To perform and calculate the standard curve/equation of GHSA, 2 µL of purified GHSA at concentrations between 0.1 and 5 mg/mL was mixed with 10 µL GO-aptasensor complex solution and 188 µL electrolyte solution (5 mM K_3_Fe(CN)_6_ and 10 mM PBS). Square wave voltammetry (SWV) and cyclic voltammetry (CV) were then performed. The SWV parameters were set at potential range of −0.5 to 1.0 V, frequency of 25 Hz, step potential of 5 mV, and amplitude of 20 mV. For CV measurement, the parameters were set at potential range of −0.5 to 0.8 V, scan rate of 100 mV/s, and step potential of 2.44 mV. Then, the GHSA concentrations and electrochemical signals were plotted, and a standard curve was calculated. For GHSA determination in human serum, 2 µL human serum was added to the reaction mixture, instead of the purified GHSA, and the GHSA concentrations were calculated based on the previous standard equation. The overall electrochemical measurements are shown in Figure 1.

### 2.5. Fluorescence Measurement

To confirm the performance of the electrochemical measurement of the GO-aptasensor for GHSA detection, the modified G8 aptamer was 5′-end labeled with a fluorescence molecule (FAM), and the fluorescence intensities were measured following a modified protocol from previous reports [19,24,25]. Briefly, 2 µL of purified GHSA or clinical samples was mixed with 10 µL of GO-aptasensor complex solution and 188 µL of electrolyte solution (5 mM K_3_Fe(CN)_6_ and 10 mM of PBS). After incubation at room temperature in the dark for 30 min, the fluorescence signal of the mixtures was measured at 495/520 nm using a portable fluorometer (Quantus; Promega, Madison, WI, USA). Then, the fluorescence signals and GHSA concentrations were plotted, and the standard equation was calculated.

## 3. Results and Discussion

### 3.1. Electrochemical Characterization

**Figure 2:**
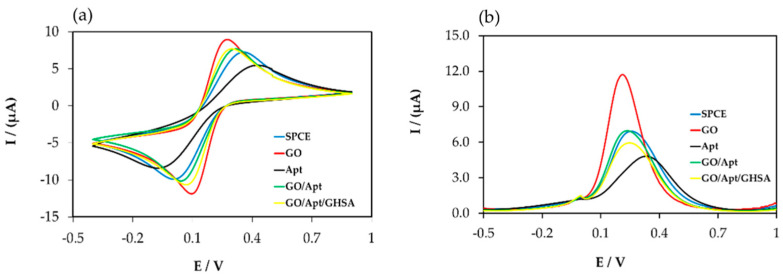
Electrochemical data for the components in the developed electrochemical aptasensor platform for GHSA detection using cyclic voltammetry (CV) measurement (**a**) and square wave voltammetry (SWV) measurement (**b**). The blue line (SPCE) represents the signal from the bare electrode, the red line (GO) represents the signal after adding free GO on the electrode surface, the black line (Apt) represents the signal after adding free aptamer on the electrode surface, the green line (GO/Apt) represents the signal after adding the GO-aptamer complex on the electrode surface, and the yellow line (GO/Apt/GHSA) represents the signal after adding the mixture of the GO-aptamer complex and GHSA.

Graphene-based nanomaterials are used as transducers in biosensors and are therefore involved in converting the interactions between the receptor and target molecules into detectable measurements [26]. GO enhances biosensor efficacy by using its advantageous properties, such as large surface area, electrical conductivity, and high electron transfer rate. In addition, reduced graphene oxide (rGO) exhibits high electrical conductivity, while GO is known to be an electrically insulating material. Therefore, in this study, we used GO and Fe(CN)_6_^3−^, which is a well-known electrochemically reversible redox couple, coupled with a GHSA-specific aptamer to develop an immobilization-free electrochemical aptasensor to detect GHSA in clinical samples. Both free GOs and GOs in the GO-aptasensor complex were adsorbed on the electrode surface, increasing the current signal via the electron transfer-enhancing properties of GO and the number of active sites for the Fe^3−^/Fe^4−^ redox reaction. The modified G8-aptamer adsorbed on the GO surface owing to the robust π-π interactions between GO and nitrogenous bases of the DNA aptamer. The formation of GO/Apt/SPCE hindered the electron transfer of Fe(CN)_6_^3−/4−^. Therefore, characteristic reduction of the electrochemical signal was observed. In the presence of GHSA, the aptamer detached from the GO surface and bound to GHSA; thus, the free GOs were subsequently deposited on the electrode surface, leading to enhancement of the electrochemical signal. In contrast, in the absence of GHSA, the aptamer remained attached on the GO surface; thus, the whole GO-aptamer complex was deposited on the electrode surface, leading to a smaller enhancement in the electrochemical signal compared with the former conditions. The electrochemical data (ΔI, I_0_, and I_1_) were then obtained, as detailed in Figure 2. ΔI was calculated as ΔI = I_0_ − I_1_, where ΔI is the current change between the signal current of the bare electrode; after adding each sample, I_0_ is the signal current of the bare electrode; and I_1_ is the signal current of the electrode after adding each sample.

The results showed that a greater increase in the signal was observed when the electrode was randomly adsorbed with the free GOs (red line), owing to the large surface area and high conductivity, leading to a larger electrode surface for charge transfer and a greater reduction signal. The smallest signal was observed when the electrode surface interacted with the free aptamer because the negatively charged phosphate backbone of the DNA aptamer hampered electron transfer between the electrode surface and electrolyte solution (black line). In the case of the GO-aptamer complex, an increase in the signal was observed when the complex was adsorbed on the electrode surface (green line). However, in the presence of GHSA, owing to the stronger binding of the aptamer and GHSA compared with the aptamer and GO, the GO was released from the GO-aptamer complex and subsequently adsorbed on the electrode surface, leading to a slightly increased signal (yellow line) owing to the favorable electron transfer of Fe(CN)_6_^3−/4−^. Although similar trends in electrochemical data were observed for both CV (Figure 2a) and SWV measurements (Figure 2b), a greater signal change was observed in the SWV measurements. Therefore, we performed experiments using only SWV measurements.

**Figure 3:**
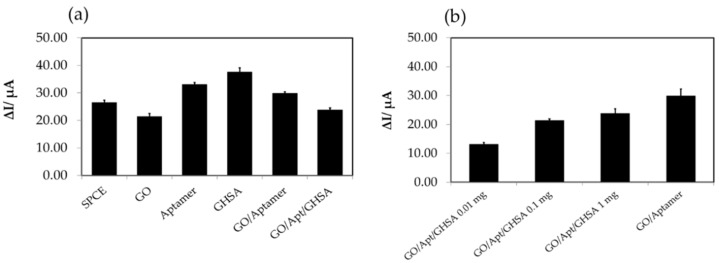
(**a**) The electrochemical characteristics of the components in the developed electrochemical aptasensor using unlabeled aptamer. (**b**) The current changes (ΔI) of different GHSA concentrations using the unlabeled aptasensor. The SPCE bar represents the ΔI of the bare electrode, the GO bar represents ΔI after adding free GO on the electrode surface, the aptamer bar represents ΔI after adding free aptamer on the electrode surface, the GHSA bar represents ΔI after adding GHSA on the electrode surface, the GO/aptamer bar represents ΔI after adding the GO-aptamer complex on the electrode surface, and the GO/Apt/GHSA bar represents ΔI after adding the mixture of GO-aptamer and GHSA on the electrode surface.

To confirm that the fluorescence molecule on the fluorescence-labeled aptamer did not interfere with the electrochemical signal, the same protocols were performed to determine the current response of the unlabeled modified G8 aptamer. The results showed similar electrochemical characteristics in which the lowest current change was observed for free GO and high current changes were observed for free aptamer and free GHSA (Figure 3a). In addition, the current change depended on the GHSA concentration, as shown in Figure 3b. These results indicated that the fluorescence molecule (FAM) on the fluorescence-labeled aptamer does not significantly interfere with the electrochemical characteristics of the developed aptasensor system. In addition, the developed aptasensor has the potential for GHSA concentration analysis.

### 3.2. Fluorescence Characterization of the Developed Aptasensor Components

**Figure 4:**
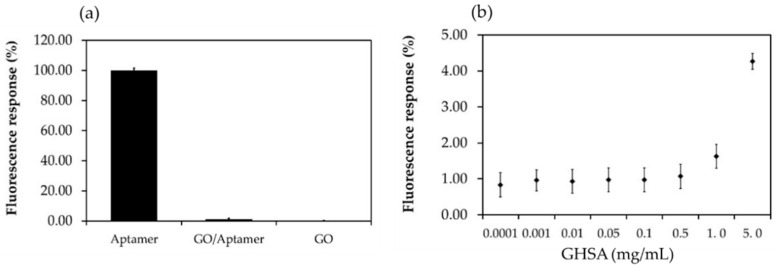
Percent fluorescence response of the components in the electrochemical aptasensor system. (**a**) Percent fluorescence response of the fluorescence-labeled aptamer (Aptamer), GO-aptamer complex (GO-aptamer), and free GO (GO). (**b**) Correlation between the percent fluorescence response and GHSA concentration (0.0001–5 mg/mL). The fluorescence signals were measured at an excitation wavelength of 480 nm and emission wavelength of 520 nm.

Next, we aimed to confirm the interaction of the modified G8 aptamer on the GO surface and the binding of the aptamer with GHSA in the electrochemical sensing system. The same components in the developed aptasensor system were used to measure the fluorescence signal. Because the fluorescence molecule used in this study did not interfere with the electrochemical system, as demonstrated in the previous section, in this experiment, the fluorescence-labeled aptamer was used. After the fluorescence intensities were measured, the percent fluorescence responses from all components were calculated and compared. As expected, the highest percent fluorescence response was observed for the fluorescence-labeled aptamer and that the lowest percent fluorescence response was observed for free GO. In addition, the fluorescence signal was almost completely quenched after incubation of the fluorescence labeled aptamer and the GO owing to the interaction of nitrogenous bases of the DNA aptamer with the GO surface (π-π stacking), leading to inhibition of fluorescence resonance energy transfer (FRET) as shown in Figure 4a. After mixing of GHSA and the GO–aptamer complex, the fluorescence signal was recovered owing to the detachment of the aptamer from the GO surface and subsequent binding of the aptamer and GHSA, leading to the FRET reaction. The results also demonstrated that the percent fluorescence response was dependent on the concentration of GHSA (Figure 4b), similar to previous electrochemical results reported by Apiwat et al. [19] and Chawjiraphan et al. [24,25]. These results support the interactions of the modified G8 aptamer on the GO surface and the binding of the aptamer with GHSA in the electrochemical sensing system.

### 3.3. Performance of the Developed Electrochemical Aptasensor

**Figure 5:**
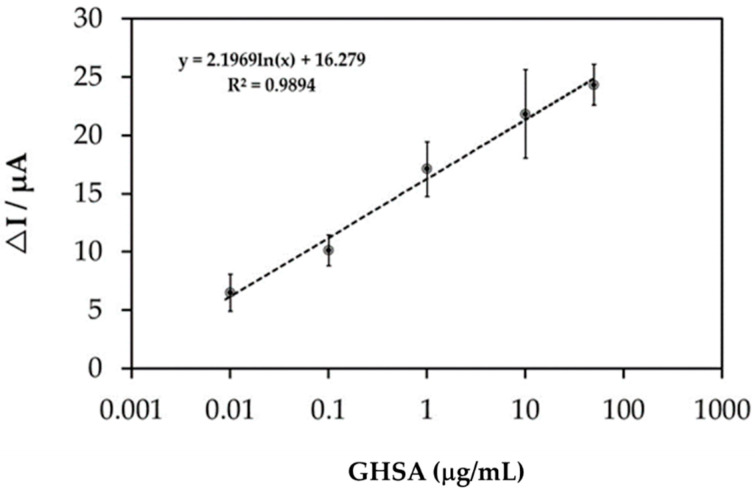
The calibration curve of the GHSA concentration on a base-10 logarithmic scale, for which y = 2.1969ln(x) + 16.279 and R^2^ = 0.9894. ΔI represents the current change.

The immobilization-free electrochemical aptasensor reaction in the presence of various GHSA concentrations (0.01–50 µg/mL) was studied using SWV measurements (Figure S3), and ΔI values were calculated. Then, ΔI values were plotted against GHSA concentrations, and the standard curve/equation was analyzed (Figure 5). The results showed that ΔI increased as a function of GHSA concentration with a base-10 logarithmic scale, where y = 2.1969ln(x) + 16.279 and R^2^ = 0.9894, where y represents the ΔI value, x represents the GHSA concentration and R^2^ represents coefficient of determination value.

The calibration curve of GHSA exhibited a base-10 log scale correlation of ΔI and plotted GHSA concentrations from 0.01 to 50 µg/mL with an R^2^ value of 0.9894. The limit of detection (LOD) was calculated using the equation LOD = 3SD/m, where SD is the standard deviation for the blank (peak current at 0 M GHSA) and m is the slope of the calibration curve [27]. The obtained LOD was 8.70 ng/mL, which is lower than almost all LODs reported in previous studies (Table 2). Apart from the lower LOD, the developed electrochemical aptasensor has the shortest processing time (30 min) because the immobilization step is not required.

### 3.4. Specificity, Reproducibility, and Stability of the Developed Electrochemical Aptasensor

The specificity of the immobilization-free electrochemical aptasensor platform was investigated using various interfering substances found in blood circulation, such as IgG, Hb, and HSA, in comparison with GHSA. The electrochemical signals were then converted to percent current normalization or percent response the following equation:

$$\%\mathrm{Current}\;\mathrm{response}=\frac{\left(\mathrm{GHSA}\;\mathrm{current}\;\mathrm{signal}-\mathrm{interference}\;\mathrm{current}\;\mathrm{signal}\right)\times 100}{\mathrm{GHSA}\;\mathrm{current}\;\mathrm{signal}}$$

**Figure 6:**
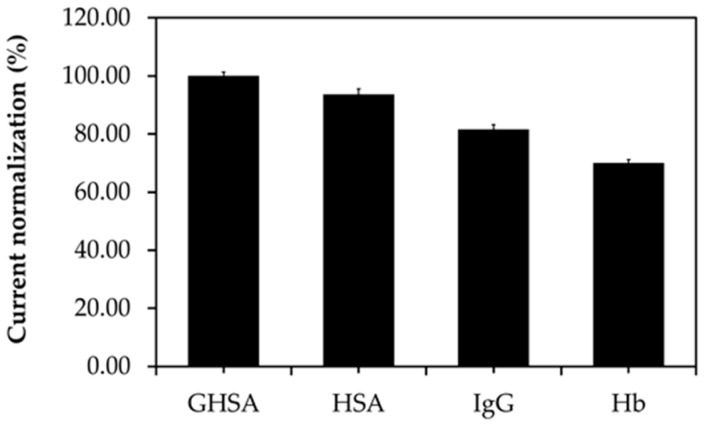
Comparison of the percent current normalization of GHSA and interfering substances present in blood circulation.

The results showed that the percent current response from GHSA was the highest (100%), whereas the percent current responses of HSA, IgG, and Hb were 52.91% ± 1.46%, 29.54% ± 1.46%, and 11.69% ± 0.93%, respectively (Figure 6). These results implied that the aptamer is weakly bound to interfering substances and shows only half of the percent current response compared with GHSA binding owing to the similar structures of HSA and GHSA [30].

**Figure 7:**
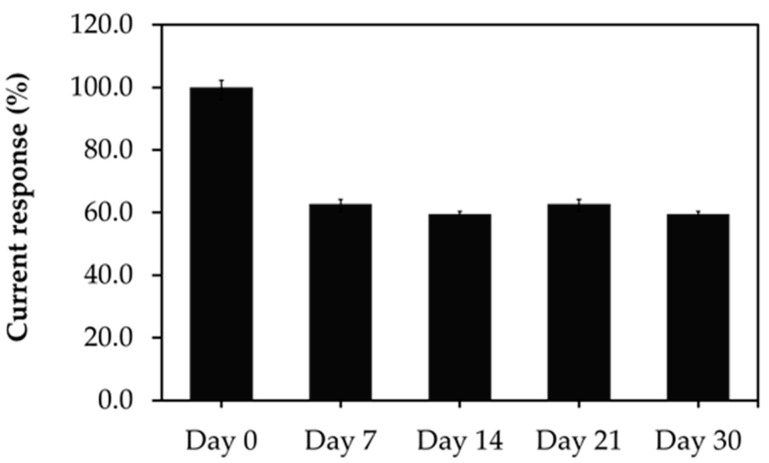
Stability of the immobilization-free aptasensor during storage at room temperature for 0, 7, 14, 21, and 30 days with a GHSA concentration of 1 µg/mL. The error bars indicate results from three individual experiments.

To evaluate the reproducibility of the developed aptasensor, five treated SPCEs were prepared under the same conditions and used for GHSA measurements in the presence of 1 µg/mL GHSA. Similar reduction signal responses were obtained with an acceptable relative standard deviation of 9.88%. Furthermore, the stability of the developed aptasensor was determined over a time range of 30 days, and the individual aptasensors were used to measure 1 µg/mL GHSA. The results showed that 60% of the current response of the aptasensor was retained after 1 week, indicating that the stability of the developed aptasensor was shorter than 7 days (Figure 7).

### 3.5. Application of the Developed Electrochemical Aptasensor for the Detection of GHSA in Clinical Samples

**Figure 8:**
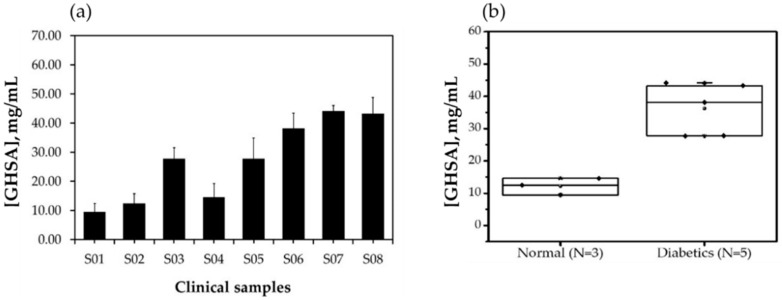
GHSA concentrations in eight human serum samples analyzed using the developed electrochemical aptasensor. Data are shown as a bar plot (**a**) and box plot (**b**). S01, S02, and S04 indicate serum samples from healthy individuals, and S03, S05, S06, S07, and S08 indicate serum samples from patients with diabetes.

Next, we aimed to assess the potential use of the developed aptasensor for GHSA detection in clinical samples. Eight human serum samples, three normal samples, and five diabetes mellitus samples were used for pre-analysis (ethical approval number: COA. MURA2019/796). The results showed that GHSA levels were significantly higher in serum from patients with diabetes than in that from patients without diabetes, as shown in Figure 8a,b and Figure S4. The results also showed that the GHSA concentrations in serum samples from patients without diabetes were in the range of 9.47 to 14.61 mg/mL, whereas those in patients with diabetes ranged from 27.71 to 44.19 mg/mL (Table 3), suggesting that the developed aptasensor has the potential to screen for diabetes. However, the ionic strength of the molecular environment affects the interactions and the electrochemical signals; therefore, this property perhaps contributes to the standard deviation of measurements in clinical samples.

## 4. Conclusions

In this study, we successfully demonstrated the concept of an immobilization-free electrochemical GO-mediated aptasensing platform for GHSA detection. The developed platform offers an alternative method for determining GHSA in clinical samples. Additionally, the developed platform exhibited good analytical performance, with an LOD of 8.70 ng/mL within the concentration range of 0.01 to 50 µg/mL. The performance of the sensor showed good sensitivity; however, the stability of the platform needs to be improved. In addition, the proposed analytical concept provides a simple protocol for one-drop analysis and rapid measurement. More importantly, fabrication and probe-immobilization processes are not required. Therefore, the proposed method demonstrates the potential of the immobilization-free electrochemical aptasensor for GHSA detection in clinical samples. The dual detection properties, such as electrochemical and fluorescence detection, may be applied for measuring GHSA concentrations in various clinical sample types having different GHSA levels. Additional studies are required to evaluate the dual detection capability of the aptasensor.

## 5. Patents

Some materials in this study have been submitted for intellectual property patents in Thailand (Petty patent; application number: 2003002006, filing date: 20 August 2020).

## Figures and Tables

**Table 1:** The nucleotide sequences of the modified G8-aptamers.

Aptamer Name	Nucleotide Sequence
Modified G8-FAM	5′-/56-FAM/-TGCGGTTCGTGCGGTTGTAGTAC-3′
Modified G8	5′-TGCGGTTCGTGCGGTTGTAGTAC-3′

**Table 2:** Comparison of the Detection Limits and Assay Times of the Developed Aptasensor in This Study and Other GHSA Detection Platforms.

Method Used	Detection Limit	Assay Time	Reference
Raman spectroscopy-based sensor	13.7 µM	1 h	[17]
Graphene-based optical aptasensor	50 µg/mL	>30 min	[19]
Electrochemical aptasensor	0.07 µg/mL	>24 h	[28]
Electrochemiluminescence	6.6 µg/mL	>2 h	[29]
Optical aptasensor	0.067 µg/mL	>2 h	[12]
Electrochemical aptasensor	3 ng/mL	>17 h	[20]
Electrochemical GO-aptasensor	8.70 ng/mL	30 min	This study

**Table 3:** Summary of GHSA concentrations analyzed by the developed electrochemical aptasensor.

Sample Source	GHSA Concentration (mg/mL)			
	Mean	SD	Minimum	Maximum
Normal	12.18	2.58	9.47	14.61
Diabetics	36.23	8.07	27.71	44.19

Cutoff = 26.83; cutoff value = (upper boundary of the current value of Group I + lower boundary of current value of Group II)/2 [31].

## Data Availability

Not applicable.

## Supplementary Materials

The following are available online at https://www.mdpi.com/2079-6374/11/3/85/s1, Figure S1: SEM images of bare Au substrate; Figure S2: Elemental mapping and EDS spectrum of GO on Au substrate; Figure S3: SWV of the developed aptasensor corresponding to the GHSA calibration curve. GHSA concentrations were 0.0001, 0.01, 0.1, 1 and 5 mg/mL; Figure S4: SWV of the developed aptasensor for GHSA analysis in serum samples (N=8). S01, S02, and S04 indicate serum samples from healthy individuals, and S03, S05, S06, S07, and S08 indicate serum samples from patients with diabetes.
