# Supplementary material for: Immobilization-Free Electrochemical Sensor Coupled with a Graphene-Oxide-Based Aptasensor for Glycated Albumin Detection
Source: Biosensors (Basel). 2021 Mar 17;11(3):85. doi: 10.3390/bios11030085 (PMC8002523; doi:10.3390/bios11030085)
Supplement: Supplementary file 1 [file biosensors-11-00085-s001.pdf]

Supplementary Materials

Immobilization-free electrochemical sensor coupled with a graphene oxide-based aptasensor for glycated albumin detection

Wassa Waiwinya[1], Thitirat Putnin[1], Dechnarong Pimalai[1], Wireeya Chawjiraphan[1], Nuankanya Sathirapongsasuti[2], and Deanpen Japrung [1,*]

[1]National Nanotechnology Center (NANOTEC), National Science and Technology Development Agency (NSTDA), Thailand Science Park, Pathumthani, Thailand

[2]Section for Translational Medicine, Faculty of Medicine Ramathibodi Hospital, Mahidol University, Bangkok, Thailand

* Correspondence: deanpen@nanotec.or.th; Tel.: +66 2564 6665

Characterization of free GO

The structural morphology of the GO nanosheet was studied using SEM and TEM. In this experiment, the GO was deposited on the Au surface for signal enhancement and better discrimination of GO sheet from electrode surface. SEM images exhibited a GO deposited on surface as shown in **Figure S1a** and **S1b**. A sheet-like GO structure was formed and consistent observed using TEM analysis as **Figure S1c** and **S1d**, suggesting the GO sheet was obtained in this work. Furthermore, the occurrence of the element on Au-surface was analysed by SEM mapping and energy dispersive X-ray spectroscopy (EDS) analysis. The result showed in **Figure S2**, which represents two typical spectra from Au-surface. The spectra of C and O atoms can be observed together with Au spectrum from surface. The EDS spectrum confirmed that the GO was successfully deposited on the substrate surface during the direct dropping aptasensor solution onto SPCE.

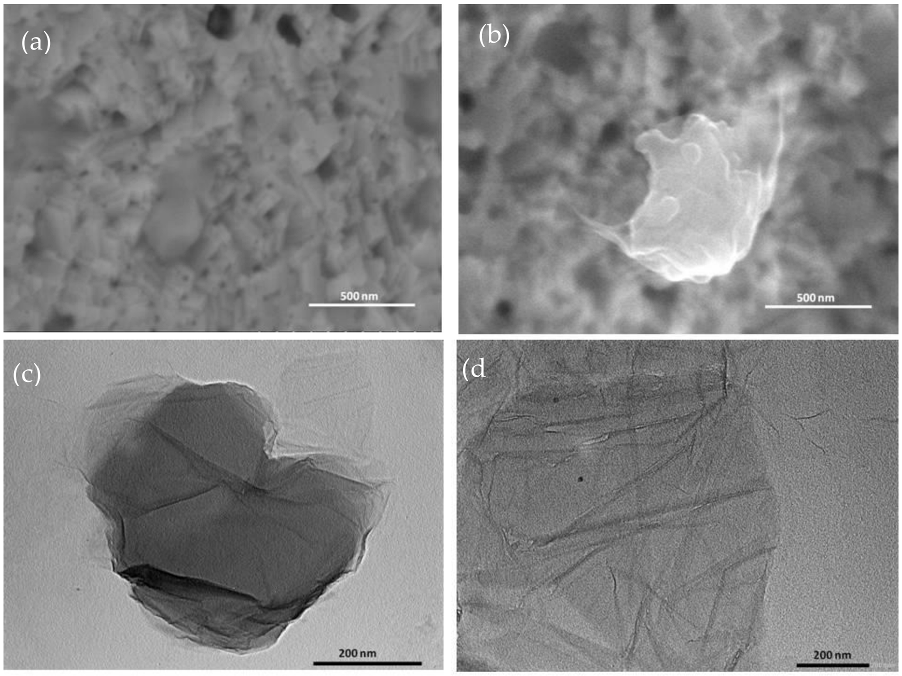

Figure S1. SEM images of bare Au substrate (**a**) and GO- Au substrate (**b**). TEM images of GO nanosheet (**c-d**).

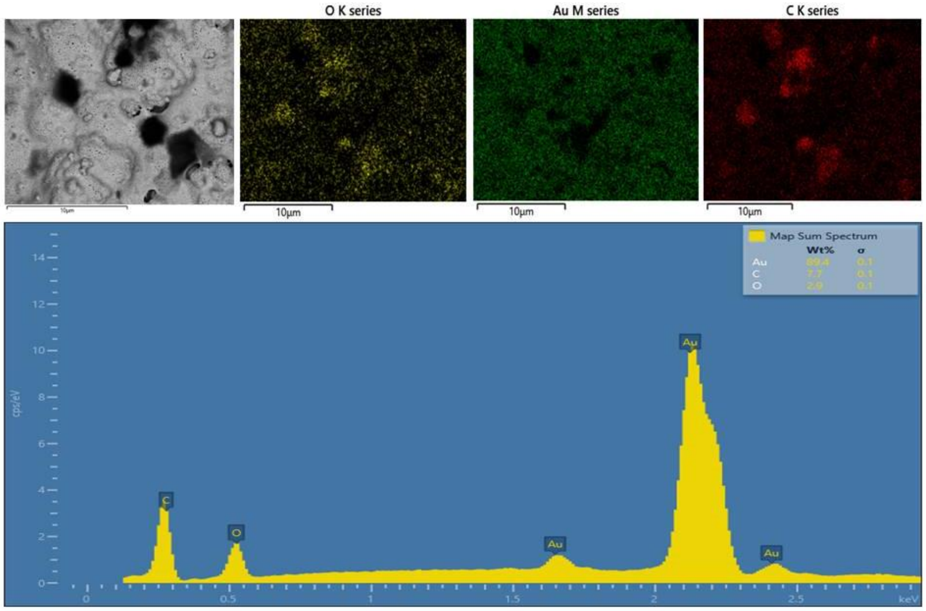

Figure S2. Elemental mapping and the EDS spectrum of GO on Au substrate.

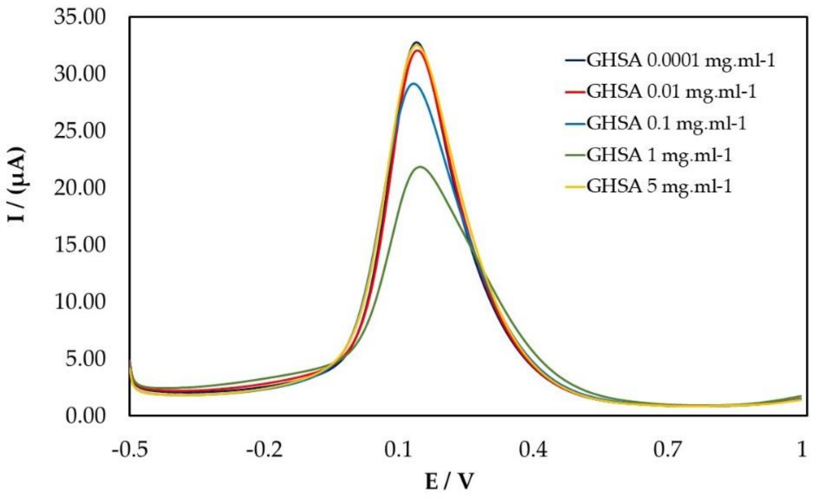

Figure S3. SWV of the developed aptasensor corresponding to the GHSA calibration curve. GHSA concentrations were 0.0001, 0.01, 0.1, 1 and 5 mg/mL.

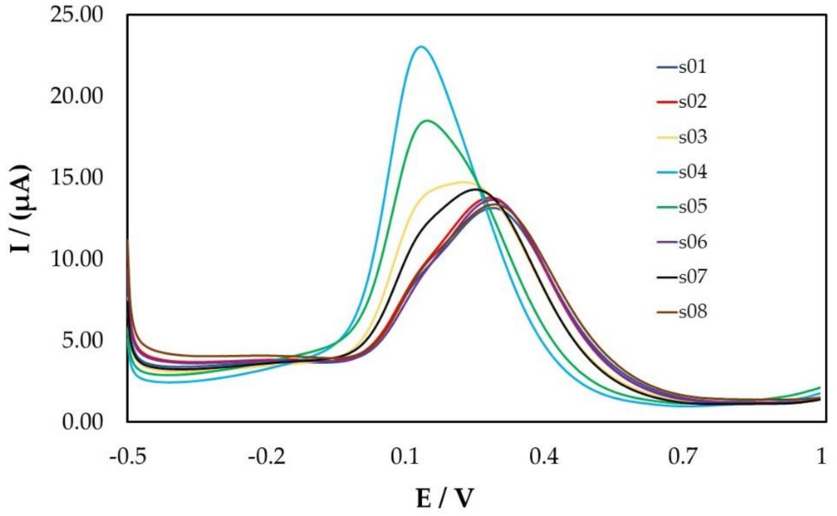

Figure S4. SWV of the developed aptasensor for GHSA analysis in serum samples (N=8). S01, S2, and S04 indicate serum samples from healthy individuals, and S03, S05, S06, S07, and S08 indicate serum samples from patients with diabetes.
